# NK Cell Reconstitution After Autologous Hematopoietic Stem Cell Transplantation: Association Between NK Cell Maturation Stage and Outcome in Multiple Myeloma

**DOI:** 10.3389/fimmu.2021.748207

**Published:** 2021-10-05

**Authors:** Ane Orrantia, Iñigo Terrén, Gabirel Astarloa-Pando, Carmen González, Alasne Uranga, Juan J. Mateos-Mazón, Juan C. García-Ruiz, Marta Riñón, Mercedes Rey, Silvia Pérez-Fernandez, Olatz Zenarruzabeitia, Francisco Borrego

**Affiliations:** ^1^ Immunopathology Group, Biocruces Bizkaia Health Research Institute, Barakaldo, Spain; ^2^ Biodonostia Health Research Institute, Hematology and Hemotherapy Service, Donostia University Hospital, Donostia-San Sebastián, Spain; ^3^ Hematological Cancer Group, Biocruces Bizkaia Health Research Institute, Hematology and Hemotherapy Service, Cruces University Hospital, Barakaldo, Spain; ^4^ Regulation of the Immune System Group, Biocruces Bizkaia Health Research Institute, Immunology Service, Cruces University Hospital, Barakaldo, Spain; ^5^ Biodonostia Health Research Institute, Immunology Service, Donostia University Hospital, Donostia-San Sebastián, Spain; ^6^ Scientific Coordination Facility, Biocruces Bizkaia Health Research Institute, Barakaldo, Spain; ^7^ Ikerbasque, Basque Foundation for Science, Bilbao, Spain

**Keywords:** NK cells, CD57, IL-15, adaptive NK cells, autologous hematopoietic stem cell transplantation, NK cell maturation, NKG2A, multiple myeloma

## Abstract

Autologous hematopoietic stem cell transplantation (autoHSCT) is a standard of care for transplant-eligible patients with multiple myeloma (MM). Among factors that influence outcome after autoHSCT, it has been suggested that the number of natural killer (NK) cells plays an important role. However, the impact that different NK cell subsets and their phenotype could have in disease progression after autoHSCT are less clear. For this reason, we have phenotypically and functionally characterized NK cells during immune system reconstitution after autoHSCT in 54 MM patients. Shortly after leukocyte recovery, an extensive redistribution of NK cell subsets occurs in these patients. In addition, NK cells undergo a profound phenotypic change characterized, among others, by their increased proliferative capacity and immature phenotype. Importantly, MM patients who showed lower frequencies of the mature highly differentiated NKG2A-CD57+ NK cell subset at +30 and +100 days after autoHSCT experienced superior progression-free survival and had a longer time to the next treatment than those with higher frequencies. Our results provide significant insights into NK cell reconstitution after autoHSCT and suggest that the degree of NK cell maturation after autoHSCT affects the clinical outcome of MM patients treated with this therapeutic strategy.

## Introduction

Autologous hematopoietic stem cell transplantation (autoHSCT) is a worldwide-established treatment option for a diverse group of hematological malignances, including multiple myeloma (MM). In fact, high-dose chemotherapy followed by autoHSCT is considered the standard of care for transplant-eligible MM patients. The achievement of an early immune recovery after autoHSCT is key for patient´s survival. Particularly, day 15 absolute lymphocyte count (ALC-15) after autoHSCT has been reported as an independent prognostic indicator in MM patients (1). Among lymphocytes, natural killer (NK) cells are the first to recover during immune reconstitution after transplantation and are recognized as the main lymphocyte subset in ALC-15 that affects outcome after autoHSCT (2).

NK cells are a crucial part of the innate immune system, being classified within the family of the innate lymphoid cells (3). One of their main features is the ability to recognize and eliminate virus-infected and malignant cells without prior sensitization. Commonly, NK cells have been classified in two major subsets, based on the differential expression of CD56 and CD16: CD56^bright^CD16^low/-^ and CD56^dim^CD16^+^ (hereafter referred to as CD56^bright^ and CD56^dim^, respectively). However, thanks to the development of pioneering experimental technologies, data published in recent years have revealed a broad diversity within NK cells (4, 5). Among factors contributing to NK cell diversity, infections by different pathogens play an important role. An expansion of a NK cell subset lacking CD56 expression, known as CD56^neg^ (CD56^neg^CD16^+^), has been widely described after human immunodeficiency virus infection (6). Furthermore, human cytomegalovirus infection (CMV) drives an expansion of NK cells that express the activating receptor NKG2C, usually known as adaptive NK cells (7). In autoHSCT and allogeneic hematopoietic stem cell transplantation (alloHSCT), both an early human CMV reactivation and/or adaptive NK cell expansion have been associated with reduced relapse (8–10). Nevertheless, few studies have performed a comprehensive analysis of how different NK cell subsets and their phenotype change during NK cell reconstitution after autoHSCT and the association of these alterations with the clinical outcome of these patients.

In the present study, we performed an extensive phenotypic and functional analysis and we investigated the frequency of NK cell subsets during their reconstitution after autoHSCT in MM patients. Early after leukocyte recovery, a complete NK cell subset redistribution occurs in these patients. NK cells undergo a profound phenotypic change characterized, among other things, by their increased proliferative capacity and immature phenotype. Interestingly, MM patients that exhibited lower frequencies of the terminally differentiated NKG2A-CD57+ NK cells at +30 and +100 days after autoHSCT experienced superior progression-free survival (PFS) and had longer time to next treatment (TTNT) than those who had higher frequencies of that NK cell subset. Our data provide an expanded insight into NK cell reconstitution after autoHSCT and highlight the effect that their degree of differentiation may have on the outcome of MM patients who have undergone an autoHSCT, which could help to further individualize and improve this therapy.

## Materials and Methods

### Patients’ Characteristics and Study Design

54 patients from Cruces University Hospital and Donostia University Hospital, who suffered from MM and received autoHSCT were included in the study. Clinical data of the patients can be found in **Table 1**. Blood samples were taken from patients at six different time points. Sample 1 (S1) was collected the day before the start of the conditioning treatment; sample 2 (S2) was collected after leukocyte recovery (>1000 leukocytes/µL) (median: 13 days after autoHSCT, range: 10-25 days); sample 3 (S3) was collected 30 days after cell infusion; sample 4 (S4) was collected 100 days after cell infusion; sample 5 (S5) was collected 180 days after cell infusion and sample 6 (S6) was collected 365 days after cell infusion. Sample collection was carried out through the Basque Biobank for Research (http://www.biobancovasco.org), which complies with the quality management, traceability and biosecurity, set out in the Spanish Law 14/2007 of Biomedical Research and in the Royal Decree 1716/2011. The study was approved by the Basque Ethics Committee for Clinical Research (BIO14/TP/003 and PI+CES+INC-BIOEF 2017-03). All subjects provided written and signed informed consent in accordance with the Declaration of Helsinki.

**Table 1:** Patients´ characteristics.

		n (%)
**Gender**	Male	30 (55.6%)
	Female	24 (44.4%)
**Myeloma classification: ISS**	ISS 1	23 (42.6%)
	ISS 2	16 (29.6%)
	ISS 3	14 (25.9%)
**Myeloma classification:**	I-A	6 (11.1%)
**Durie-Salmon Staging System**	II-A	19 (35.2%)
	II-B	2 (3.7%)
	III-A	22 (40.7%)
	III-B	5 (9.3%)
**Mobilization regimen**	G-CSF	52 (96.3%)
	G-CSF + Plerixafor	2 (3.7%)
**Conditioning regimens**	Melphalan 140	2 (3.7%)
	Melphalan 200	47 (87.1%)
	BUMEL	5 (9.3%)
**CMV serostatus**	CMV+	48 (88.9%)
	CMV-	5 (9.3%)
**Pre-autoHSCT response**	CR	13 (24.1%)
	VGPR	22 (40.7%)
	PR	17 (31.5%)
	SD	2 (3.7%)
**Post-autoHSCT response (+100 days)**	CR	25 (46.3%)
	VGPR	19 (35.2%)
	PR	9 (16.7%)
	SR	1 (1.9%)
**Maintenance regimen or consolidation**	Yes	29 (53.7%)
**regimen**	No	25 (46.3%)
**Disease progression**	Yes	29 (53.7%)
	No	25 (46.3%)
**Alive**	Yes	45 (83.3%)
	No	9 (16.7%)
		**Median (interquartile range)**
**Age**		62 (33–73)
**Infused CD34+ cells (x10^6^ cells/kg)**		2.94 (1.9-6.86)
**Months to disease progression**		21 (2–46)
**Months to death**		29 (8–57)
**Months to next treatment line**		25.5 (3–50)

ISS indicates International Staging System; G-CSF, granulocyte colony-stimulating factor; CMV, cytomegalovirus; BUMEL, busulfan-melphalan; CR, complete remission; VGPR, very good partial response; PR, partial response; SD, stable disease; SR, stable response.

### Sample Preparation

Plasma and peripheral blood mononuclear cells (PBMCs) were obtained from blood samples. PBMCs were isolated by Ficoll (GE Healthcare) density gradient centrifugation, cryopreserved in heat-inactivated Fetal Bovine Serum (FBS) (GE Healthcare Hyclone) containing 10% Dimethylsulfoxide (DMSO) (Thermo Scientific Scientific) and stored in liquid nitrogen until they were used. Plasma samples were stored at -80⁰C until they were used.

Prior to flow cytometry experiments, cryopreserved PBMCs were thawed at 37⁰C in a water bath and washed twice with RPMI 1640 medium supplemented with L-Glutamine (Lonza). Then, cells were incubated for 1 hour at 37⁰C and 5% CO_2_ with 10U DNase (Roche) in R10 medium (RPMI 1640 medium containing GlutaMAX from Thermo Fisher Scientific, 10% FBS and 1% Penicillin-Streptomycin (P-S) from Thermo Fisher Scientific). Afterwards, cells were washed once, resuspended in NK cell medium (RPMI 1640 medium containing GlutaMAX, 10% FBS, 1% P-S, 1% MEM Non-Essential Amino Acids Solution and 1% Sodium Pyruvate, both from Thermo Fisher Scientific), filtered using 70μm cell strainers and counted just before further use.

### NK Cell Absolute Number Determination

The absolute number of NK cells was measured in each blood sample. NK cells were defined as CD45+, CD3-, CD56+ and/or CD16+. The following clinical grade fluorochrome conjugated monoclonal antibodies (mAbs) were used: FITC anti-CD16 (CLB/FcGran1), PE anti-CD56 (MY31), PerCP-Cy5.5 anti-CD3 (SK7) and V450 anti-CD45 (2D1) from BD Biosciences. The absolute number per µL of blood was calculated based on the following formula: (percentage of NK cells within lymphocyte gate X absolute number of lymphocytes)/100. The absolute number of lymphocytes was obtained from the hemogram.

### Phenotypic Analysis

For phenotypic analysis of NK cells, PBMCs were first stained with LIVE/DEAD Fixable Aqua Dead Cell Stain Kit (Invitrogen) reagent to exclude dead cells, following manufacturer´s recommendations. Afterwards, cells were washed with PBS containing 2.5% bovine serum albumin (BSA) (Sigma-Aldrich) and extracellular staining was performed. For that, cells were incubated for 30 minutes on ice in the dark with the following fluorochrome conjugated mAbs: BV421 anti-CD56 (NCAM 16.2), BV510 anti-CD3 (UCHT1), BV510 anti-CD14 (MφP9), BV510 anti-CD19 (SJ25C1) from BD Bioscience; PE anti-NKG2C (134591) from R&D Systems; PE-Vio770 anti-NKp80 (4A4.D10), APC-Vio770 CD57 (REA769) and APC-Vio770 CD69 (REA824) from Miltenyi Biotec and APC anti-NKG2A (Z199) from Beckman Coulter. Cells were then washed again with PBS containing 2.5% BSA and fixed and permeabilizated prior to intracellular staining. Two different intracellular staining protocols were used. For “phenotype panel 1” (see **Supplementary Table 1**), PBMCs were fixed by incubating them with 4% paraformaldehyde (PFA) (Sigma-Aldrich/Merck) for 15 minutes on ice. Next, cells were washed twice with PBS containing 2.5% BSA and permeabilized by incubating with 1x BD Perm/Wash Buffer (BD Bioscience) for 15 minutes at room temperature (RT). Then, cells were incubated for 30 minutes on ice with FITC anti-FcϵRIγ (Merck) antibody. On the other hand, for “phenotype panel 2” (see **Supplementary Table 1**), cells were fixed and permeabilized with Foxp3/Transcription Factor Staining Buffer Set (eBioscience) following manufacturer´s recommendations. Then, cells were incubated for 30 minutes at RT with the following mAbs: FITC anti-Ki67 (20Raj1, Invitrogen) and PE anti-Granzyme B (GB11, BD Bioscience). In both intracellular staining protocols, after the incubation with the corresponding antibodies, cells were washed with the corresponding permeabilization buffer and resuspended in PBS. Samples were then acquired in a MACSQuant Analyzer 10 flow cytometer (Miltenyi Biotec). Flow cytometry panels used to study NK cell phenotype are shown in **Supplementary Table 1**.

### Functional Assay

For functional assays, after thawing and counting the cryopreserved PBMCs, cells in NK cell culture medium were plated at 0.5 x 10^6^ cells/well in 48-well plates. PBMCs were then primed with 10 ng/mL of recombinant human (rh) interleukin (IL)-15 (Miltenyi Biotec) and cultured for 20-21 hours. Then, for cytokine stimulation, 10 ng/mL of rhIL-12 (Miltenyi Biotec) and 50 ng/mL of rhIL-18 (MBL International) were also added in addition to rhIL-15. For target cell stimulation, 721.221 cells were added after the 20-21 hours of culture with rhIL-15 at Effector:Target (E:T) 1:1 ratio (0.5x10^6^ PBMCs and 0.5x10^6^ 721.221 cells). Then, IL-12+IL-15+IL-18 and 721.221 stimulated PBMCs were cultured for 5 additional hours. PE anti-CD107a (REA792, Miltenyi Biotec) was added at the start of the co-culture period to all the conditions and GolgiStop (monensin) and GolgiPlug (brefeldin A) protein transport inhibitors (BD Biosciences) were added after 1 hour for the rest of the incubation time following manufacturer’s protocol. Afterwards, viability and extracellular staining was performed as explained in the *Phenotypic Analysis* section. Next, cells were fixed with 4% PFA and permeabilized using 1x BD Perm/Wash Buffer. Then, cells were incubated for 30 minutes on ice with PerCP-Cy5.5 anti-interferon (IFN)γ (B27, BD Biosciences) and APC anti-tumor necrosis factor (TNF) (MAb11, BioLegend). Samples were acquired in a MACSQuant Analyzer 10 flow cytometer. The flow cytometry panel used to study NK cell functionality is shown in **Supplementary Table 1**. The percentage of NK cells positive for CD107a, IFNγ and TNF was calculated after subtracting the non-stimulus condition. 721.221 cell line was cultured in NK cell medium supplemented with 5 µg/mL Plasmocin (InvivoGen). 721.221 cell line was routinely tested for mycoplasma infection with Venor GeM Classic detection kit (Minerva Biolabs).

### Measurement of IL-15 Levels

For the measurement of IL-15 plasma levels, the Human IL-15 Quantikine ELISA Kit (R&D Systems) was used, following manufacturer’s recommendations. IL-15 plasma levels were measured in S1, S2, S3 and S4 patients´ samples. The optical density was determined using Varioskan Flash fluorimeter (Thermo Fisher Scientific) set to 450nm.

### Statistics

Flow cytometry data were analyzed using FlowLogic v.7.3 (Inivai Technologies) software. GraphPad Prism v.9 was used for graphical representation and statistical analysis. Data were tested for normal distribution with Shapiro-Wilk normality test. If data were normally distributed, Student´s t test for paired values was used to determine significant differences. Non-normal distributed data were compared with Wilcoxon matched-pairs signed rank test. Statistical analysis were done by comparing S2 with the rest of the samples. Data were represented as boxplots with the median and 25–75th percentiles, as violin plots with the median and quartiles or as bar plots as mentioned in each figure legend.

For bivariate analysis, normality of continuous variables was tested with Shapiro-Wilks test. Mean and standard deviation (SD) was presented when the variable followed a normal distribution or median interquartile range (IQR) otherwise. The Student´s t test or Mann-Whitney U test were used to compare continuous variables between groups. Qualitative variables were compared using the Chi-square or Fisher exact test. Correlation plots between variables were calculated and visualized as correlograms. Spearman´s Rank Correlation coefficient was indicated by square size and heat scale. Bivariate analysis was performed using Cox proportional hazard regression models. Survival curves were estimated with Kaplan-Meier method and compared by log-rank test. All the analysis were performed with R (version 4.0.4): A language and environment for statistical computing. R Foundation for Statistical Computing, Vienna, Austria

Data from ELISA experiments was processed following manufacturer´s recommendations. GraphPad Prism software was used to fit the standard curve with nonlinear regression and log-log line model.

## Results

### NK Cells Recovery Is Achieved 30 Days After autoHSCT

**Figure 1:**
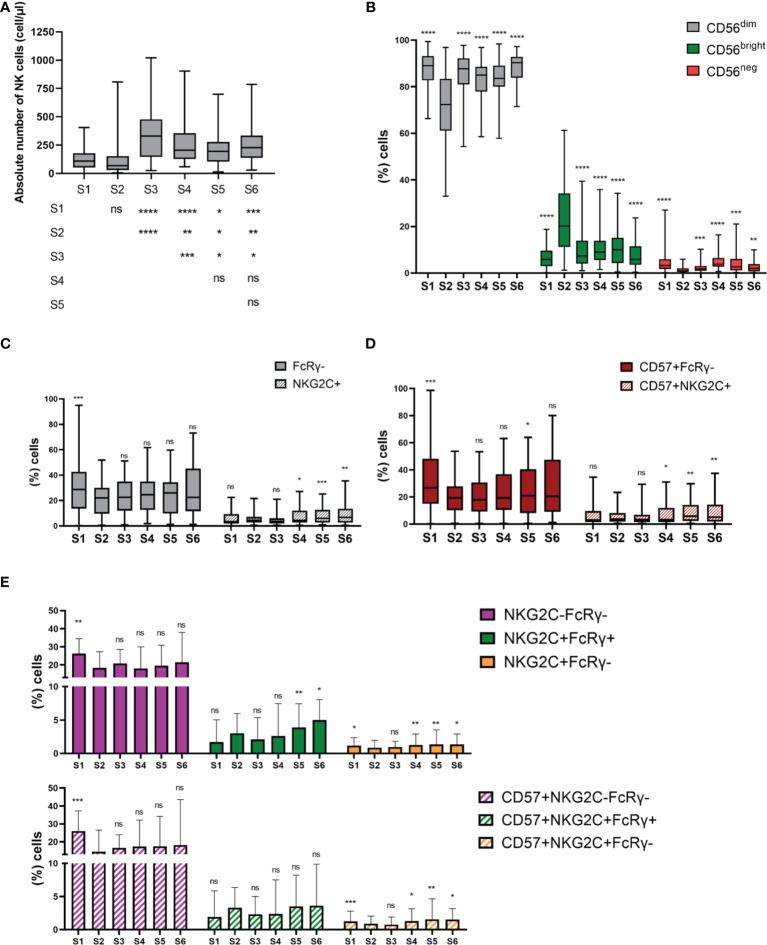
NK cell subsets distribution is altered during NK cell reconstitution after autoHSCT. **(A)** Boxplot graphs showing the absolute number of NK cells at the six studied time points (S1-S6) (S1 n=52; S2 n=45; S3 n=43; S4 n=41; S5 n=33; S6 n=35). **(B)** Boxplot graphs showing the percentage of CD56^dim^, CD56^bright^ and CD56^neg^ NK cells. (S1 n=54; S2 n=45; S3 n=45; S4 n=43; S5 n=37; S6 n=37) **(C)** Boxplot graphs showing the percentage of CD56^dim^FcRγ- and CD56^dim^NKG2C+ adaptive NK cells (S1 n=54; S2 n=45; S3 n=44; S4 n=43; S5 n=37; S6 n=37). **(D)** Boxplot graphs showing the percentage of FcRγ- and NKG2C+ adaptive NK cells within CD56^dim^CD57+ NK cells (S1 n=54; S2 n=45; S3 n=44; S4 n=43; S5 n=37; S6 n=37). **(E)** Bar graphs showing the percentage CD56^dim^NKG2C-FcRγ-, CD56^dim^NKG2C+FcRγ+ and CD56^dim^NKG2C+FcRγ- adaptive NK cells (upper panel) and NKG2C-FcRγ-, NKG2C+FcRγ+ and NKG2C+FcRγ- adaptive NK cells within CD56^dim^ CD57+ NK cells (bottom panel) (S1 n=54; S2 n=45; S3 n=44; S4 n=43; S5 n=37; S6 n=37). Boxplots show the median and 25–75th percentiles, and the whiskers denote lowest and highest values. Bar graphs show the median with the interquartile range. Significance of data was determined by comparing each sample with sample S2, except in **(A)**. *p < 0.05, **p < 0.01, ***p < 0.001, ****p < 0.0001 and ns, no significant (t test for paired values or Wilcoxon matched-pairs signed rank test).

We first studied NK cell recovery of 54 MM patients after autoHSCT. Although not significant, a decrease in the absolute number of NK cells was noticed from S1 to S2 [(median S1: 108.2 cell/µl (range: 1 – 406 cell/µl) *vs* S2: 69.00 cell/µl (range: 4 - 806.5 cell/µl)]. Afterward, a significant increase was observed at S3, followed by a decrease at S4. The absolute number of NK cells was maintained at the same levels after that; although, the values were significantly higher than what was observed in S1 and S2 (**Figure 1A**). Thus, these results suggest that the recovery of NK cells is not completely achieved until 30 days after autoHSCT (S3).

### NK Cell Subset Distribution Is Altered at Leukocyte Recovery

We next studied the distribution of different NK cell subsets. NK cells were identified as viable CD3-/CD14-/CD19-/NKp80+ cells (11, 12). Then, based on the expression of the CD56 marker, two major NK cell subsets were defined: CD56^bright^ and CD56^dim^ (**Supplementary Figure 1A**). CD56^dim^ NK cells were the main subset at all time points. However, their frequency significantly decreased at S2, while the frequency of CD56^bright^ greatly increased. Nevertheless, this redistribution of NK cell subsets was not maintained over time (**Figure 1B**). We observed no differences in terms of the frequency of CD56^bright^ and CD56^dim^ NK cells at S2 between patients having a leukocyte recovery time period of <13 days *vs* ≥13 days (**Supplementary Figures 2A, B**). In addition, we studied the distribution of a third NK cell subset known as CD56^neg^ NK cells. We have recently described an expansion of this subset in MM patients (11). In the case of autoHSCT setting, the frequency of CD56^neg^ NK cells significantly dropped at S2 and showed variations over the studied period (**Figure 1B**). These results suggest that NK cell subset distribution is altered at leukocyte recovery, but resembles pre-transplant distribution 30 days after autoHSCT.

We next used CD57, NKG2C and FcϵRγ markers to study the different adaptive subpopulations within CD56^dim^ NK cells and within CD56^dim^CD57+ NK cells (**Supplementary Figure 1B**). First, we determine the frequencies of FcϵRγ-, CD57+FcϵRγ-, NKG2C+ and CD57+NKG2C+ adaptive NK cell subpopulations. Results showed that adaptive NK cells lacking the expression of FcϵRγ were more abundant than the NKG2C+ ones (**Figures 1C, D**). In addition, the percentage of FcϵRγ- adaptive cells was reduced at S2 and maintained after that (**Figure 1C**). In contrast, no significant differences in the frequency of NKG2C+ NK cells were noticed from S1 to S3. However, an expansion of these subsets was observed at S4 and their frequency continued elevated thereafter (**Figure 1C**). Next, we analyzed the co-expression of NKG2C and FcϵRγ markers identifying four different populations. NKG2C-FcϵRγ+ population represents conventional NK cells and was therefore not included in the analysis. Regardless of CD57 expression, the main population at all time points were those with the NKG2C-FcϵRγ- phenotype. Moreover, the frequency of both CD57+NKG2C-FcϵRγ- and NKG2C-FcϵRγ- populations was reduced at S2 and maintained low after that (**Figure 1E**). In contrast, the frequency of NKG2C+FcϵRγ+ and CD57+NKG2C+FcϵRγ+ was not significantly altered immediately after autoHSCT, while the frequency of NKG2C+FcϵRγ- and CD57+NKG2C+FcϵRγ- was reduced at S2 and expanded at S4 (**Figure 1E**). These results suggest a different reconstitution pattern among adaptive NK cell populations after autoHSCT.

### NK Cells Exhibit an Immature Phenotype at Leukocyte Recovery That Last for More Than 30 Days After autoHSCT

**Figure 2:**
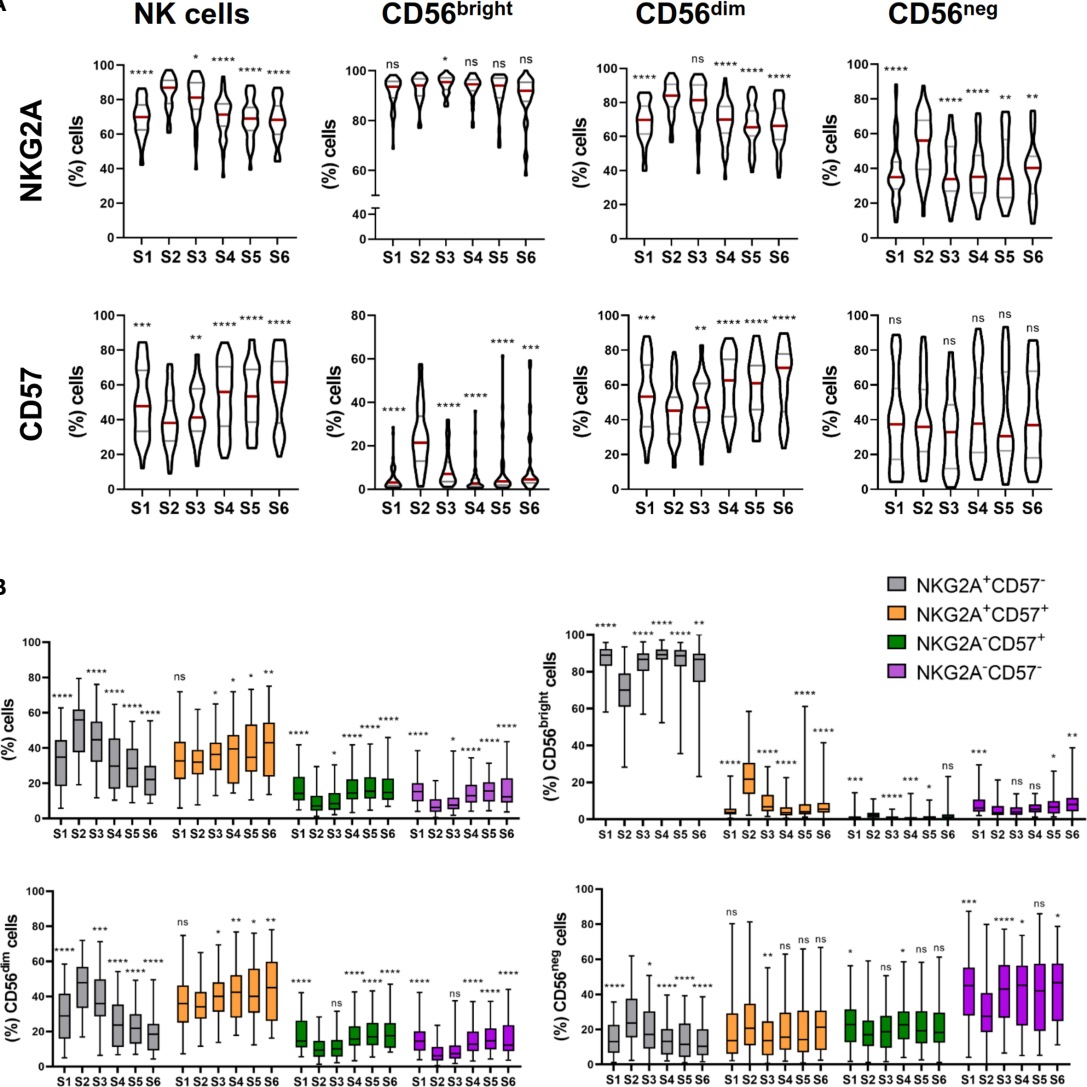
NK cells exhibit a predominantly immature phenotype shortly after autoHSCT. **(A)**, (upper panel) Violin plots showing the percentage of NKG2A+ cells within total NK cells and CD56^bright^, CD56^dim^ and CD56^neg^ NK cell subsets at the six studied time points (S1-S6). **(A)**, (lower panel) Violin plots showing the percentage of CD57+ cells within total NK cells and CD56^bright^, CD56^dim^ and CD56^neg^ NK cell subsets. **(B)** Boxplot graphs showing the percentage of NKG2A+CD57-, NKG2A+CD57+, NKG2A-CD57+ and NKG2A-CD57- within total NK cells (upper line, left) and within CD56^bright^ (upper line, right), CD56^dim^ (bottom line, left), CD56^neg^ (bottom line, right) NK cells. Violin plots show the median and the quartiles. Boxplots show the median and 25–75th percentiles, and the whiskers denote lowest and highest values (S1 n=54; S2 n=45; S3 n=45; S4 n=43; S5 n=37; S6 n=37). Significance of data was determined by comparing each sample with sample S2. *p < 0.05, **p < 0.01, ***p < 0.001, ****p < 0.0001 and ns, no significant (t test for paired values or Wilcoxon matched-pairs signed rank test).

As the frequency of CD56^bright^ NK cells notably increases at leukocyte recovery, and these cells are considered more immature than CD56^dim^ NK cells (13), we next studied the NK cell differentiation and maturation status using NKG2A and CD57 markers. While CD57 has been described to be expressed by terminally differentiated NK cells, NKG2A is expressed in earlier differentiation stages (14–16). As others have previously reported (17), the frequency of NKG2A+ NK cells increased from S1 to S2 and, although decreased at S3, remained elevated until S4 (**Figure 2A**, upper panel). In contrast, CD57+ NK cells behave the opposite way (**Figure 2A**, lower panel). The variations observed in the frequency of NKG2A+ and CD57+ cells were mainly due to an increment in the frequency of CD56^dim^NKG2A+ and CD56^neg^NKG2A+ NK cells, and a decrease in the frequency of CD56^dim^CD57+ NK cells respectively (**Figure 2A**). Surprisingly, an expansion of CD56^bright^ NK cells expressing CD57 was observed at leukocyte recovery (S2) (**Figure 2A**). However, as they are immature NK cells, CD56^bright^ NK cells are not expected to express CD57 (16).

During the final steps of differentiation, NK cells lose the expression of NKG2A while acquire CD57 (15). Therefore, we analyzed the co-expression of both markers after the autoHSCT. We found that NKG2A+CD57- NK cells were the main subset at S2, but over time, the frequency of NKG2A+CD57+ NK cells gradually increased, turning out to be the predominant subset after day 180 post-autoHSCT (S5). Moreover, the percentage of NKG2A-CD57+ NK cells also gradually augmented after the initial decrease at S2 (**Figure 2B**). When CD56^bright^, CD56^dim^ and CD56^neg^ NK cell subsets were separately analyzed we found that while within CD56^bright^ NK cells, NKG2A+CD57- cells were the most abundant subset at all time points, CD56^dim^ NK cells were mostly NKG2A+CD57- at leukocyte recovery and NKG2A+CD57+ after day 100 post-autoHSCT. In contrast, the majority of CD56^neg^ NK cells were NKG2A-CD57- at all time points (**Figure 2B**). Together, these data reveal that NK cells have a more immature phenotype at leukocyte recovery, as demonstrated by the increment in the frequency of NKG2A+CD57- cells, and that this phenotype last for more than 30 days after autoHSCT (% of NKG2A+ cells in S1 *vs* S3: p<0,0001; S1 *vs* S4: ns; S1 *vs* S5: ns; and S1 *vs* S6: ns; % of CD57+ cells in S1 *vs* S3: p = 0,0468; S1 *vs* S4: ns,; S1 *vs* S5: ns; and S1 *vs* S6: p = 0,0139; % of NKG2A+CD57- cells in S1 *vs* S3: p<0,0001; S1 *vs* S4: ns; S1 *vs* S5: ns; and S1 *vs* S6: p = 0,0072; % of NKG2A+CD57+ cells in S1 *vs* S3: ns; S1 *vs* S4: ns; S1 *vs* S5: ns; and S1 *vs* S6: p = 0,0088; % of NKG2A-CD57+ cells in S1 *vs* S3: p <0,0001; S1 *vs* S4: ns; S1 *vs* S5: ns; and S1 *vs* S6: ns; % of NKG2A-CD57- cells in S1 *vs* S3: p <0,0001; S1 *vs* S4: p = 0,0487; S1 *vs* S5: ns; and S1 *vs* S6: ns).

### NK Cell Activation, Cytotoxic Potential and Proliferation Are Modulated During NK Cell Reconstitution and Differ Among NK Cell Subsets

**Figure 3:**
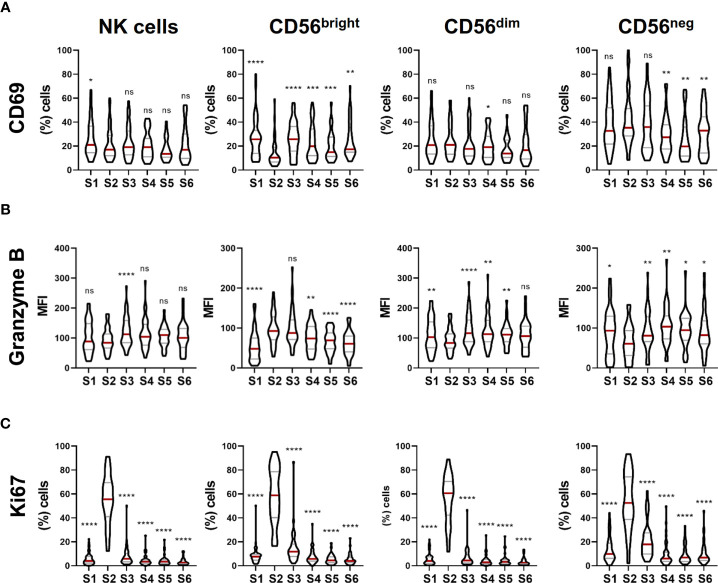
NK cell activation, cytotoxic potential and proliferation capacity are modulated during NK cell reconstitution after autoHSCT. **(A)** Violin plots showing the percentage of CD69+ cells within total NK cells and CD56^bright^, CD56^dim^ and CD56^neg^ NK cell subsets at the six studied time points (S1-S6) (S1 n=46; S2 n=37; S3 n=37; S4 n=35; S5 n=29; S6 n=29). **(B)** Violin plots showing the median fluorescence intensity (MFI) of granzyme B within total NK cells and CD56^bright^, CD56^dim^ and CD56^neg^ NK cell subsets (S1 n=54; S2 n=43; S3 n=44; S4 n=43; S5 n=35; S6 n=36). **(C)** Violin plots showing the percentage of Ki67+ cells within total NK cells and CD56^bright^, CD56^dim^ and CD56^neg^ NK cell subsets (S1 n=54; S2 n=43; S3 n=44; S4 n=43; S5 n=35; S6 n=36). Violin plots show the median and the quartiles. Significance of data was determined by comparing each sample with sample S2. *p < 0.05, **p < 0.01, ***p < 0.001, ****p < 0.0001 and ns, no significant (t test for paired values or Wilcoxon matched-pairs signed rank test).

Next, we further analyzed the phenotype of NK cells during cell reconstitution after autoHSCT. For that, we studied the activation status and the cytotoxic potential of NK cells. The analysis of the expression of CD69, an activation marker (18), showed that the frequency of CD69+ NK cells slightly decreased at leukocyte recovery, and not significant differences were noticed after that. This decrease might be principally due to a lower percentage of CD56^bright^ NK cells expressing CD69 at S2 (**Figure 3A**). On the other hand, we evaluated the cytotoxic potential of NK cells after autoHSCT by assessing the expression of granzyme B. The expression of this marker did not differ on total NK cells from S1 to S2, but it was significantly incremented at S3. When the three NK cell subsets were separately analyzed, we observed that the levels of granzyme B on CD56^bright^ NK cells were greatly increased at S2, maintained elevated at S3 and gradually reduced thereafter. Conversely, CD56^dim^ NK cells expressed less granzyme B at S2 than at S1, but the expression increased again at S3 and it was higher than at pre-autoHSCT. In the case of CD56^neg^ NK cells, a reduced expression was also observed at S2, and the expression was gradually incremented after that (**Figure 3B**). These data suggest that the activation status and the cytotoxic potential is different among the three studied NK cell subsets during NK cell reconstitution. Moreover, these data showed that the cytotoxic potential of CD56^bright^ NK cells is incremented after autoHSCT.

Finally, we used the Ki67 nuclear protein as a marker to study NK cell proliferation (19). Results showed that there was a dramatic increase in the frequency of Ki67-expressing cells in total NK cells and in the three different NK cell subsets at leukocyte recovery that was not found at other time point (**Figure 3C**), indicating that NK cells exhibit a high proliferation rate immediately after autoHSCT while the majority of them enter into a quiescent state 30 days after autoHSCT.

### NK Cells Are Functional After autoHSCT

Once phenotypic features of NK cells during their reconstitution after autoHSCT were analyzed, we next studied their functional capacities. For that, we examined degranulation and cytokine production by measuring CD107a and TNF, respectively, after stimulation with the 721.221 cell line and IFNγ production after IL-12+IL-15+IL-18 stimulation. Results revealed that, in comparison to CD56^dim^ NK cells, CD56^bright^ NK cells exhibited a trend towards an increase in their effector functions (**Supplementary Figure 3A**). No statistically significant differences were observed in the effector functions during NK cell reconstitution in any subset (**Supplementary Figure 3B**). Thus, as others have previously described (17, 20), these data suggest that the effector functions of NK cells were completely recovered early after autoHSCT.

### Elevated IL-15 Plasma Levels at Leukocyte Recovery Correlates With the Number, Proliferation Capacity and the Cytotoxic Potential of NK Cells After autoHSCT

**Figure 4:**
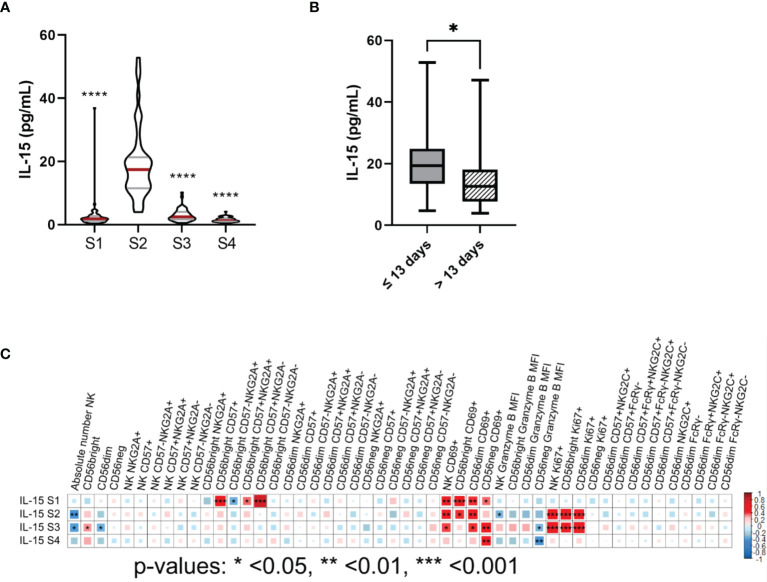
IL-15 plasma levels are elevated at leukocyte recovery and associate with the number, proliferation capacity and the cytotoxic potential of NK cells after autoHSCT. **(A)** Violin plots showing the IL-15 plasma levels (pg/mL) at the four studied time points (S1-S4) (S1 n=54; S2 n=47; S3 n=45; S4 n=42). **(B)** Boxplot graphs showing the IL-15 plasma levels (pg/mL) of patients with a time period of ≤13 days (n=30) or >13 days (n=16) between autoHSCT and S2. **(C)** Correlogram showing Spearman correlation of the indicated flow cytometry data at S2 and IL-15 plasma levels. Violin plots show the median and the quartiles. Boxplots show the median and 25–75th percentiles, and the whiskers denote lowest and highest values. Significance of data in **(A)** was determined by comparing each sample with sample S2 using Wilcoxon matched-pairs signed rank test. Data in **(B)** was compared using Mann Whitney test.*p < 0.05, **p < 0.01, ***p < 0.001, ****p < 0.0001.

IL-15 is a cytokine that plays an important role not only in proliferation, cytotoxic activity and cytokine production of NK cells, but also in their development and differentiation (21, 22). In addition, Porrata et al. showed that IL-15 levels at day 15 after autoHSCT affect survival of non-Hodking lymphoma (NHL) patients through NK cell recovery (23). Thus, we determined plasma levels of IL-15 at different time points (S1, S2, S3 and S4). Results showed a large increment of IL-15 levels at S2 (**Figure 4A**). Moreover, patients with ≤13 days between autoHSCT and S2 showed significantly higher levels of IL-15 at S2 than those with >13 days (**Figure 4B**). Nevertheless, no association was found between IL-15 levels at S2 and OS (overall survival) and PFS of these patients (data not shown).

Next, we performed correlation analysis and we found that IL-15 levels at S2 negatively correlated with absolute number of NK cells at S2. However, a positive association was observed at S2 between IL-15 levels and the percentage of Ki67+ NK cells and CD69+ NK cells. On the other hand, we found a negative correlation between IL-15 levels and the expression of granzyme B on NK cells at S2 (**Figure 4C** and **Supplementary Figures 4A–D**). Then, we categorized the patients in two groups based on their IL-15 plasma levels at S2 (median: 17.45pg/mL, range: 3.96-52.83pg/mL). We observed that patients that had higher IL-15 plasma levels showed significantly lower NK cell absolute numbers, significantly higher frequency of Ki67+ NK cells and expressed less granzyme B in NK cells at S2 (**Supplementary Figure 4E**). However, no significant differences were noticed in the percentage of CD69+ cells between groups (**Supplementary Figure 4E**). Therefore, these results suggest that IL-15 play a major role in NK cell proliferation capacity and also affect the cytotoxic potential and NK cell numbers after autoHSCT.

### The Differentiation and Maturation Status of NK Cells at 30 and 100 Days After autoHSCT Associates With the Clinical Outcome of Multiple Myeloma Patients

As NK cell count at 15 days post-autoHSCT has been defined as an independent predictor for survival after autoHSCT, we finally investigated whether a specific NK cell phenotype was associated with the clinical outcome of MM patients after autoHSCT. As shown in **Table 1**, from the 54 patients included in this study, 29 patients experience disease progression (53.7%) and 9 patients died (16.7%) during the studied period (60 months). Thus, we performed bivariate analysis for PFS, OS and TTNT, and the phenotypic data generated in the present study, but we did not find any association (data not shown). Each phenotypic variable was then dichotomized into upper (>66^th^ percentile) and middle/low (≤66^th^ percentile) groups for further analysis. However, we did not obtain any significant result when bivariate analysis was done with dichotomized variables (data not shown).

**Figure 5:**
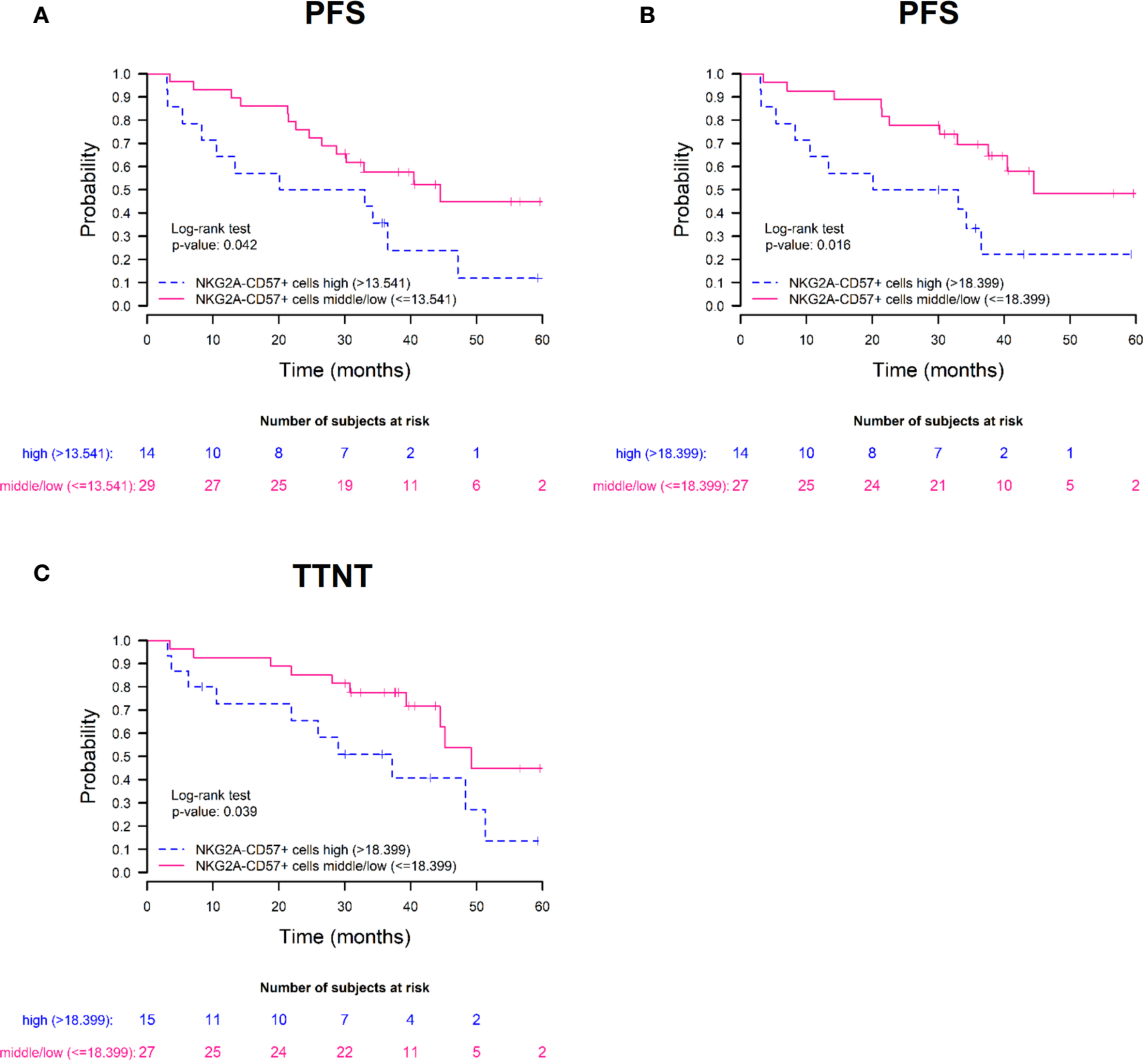
High frequencies of terminally differentiated NKG2A-CD57+ NK cells associate with poor prognosis after autoHSCT in MM patients. Survival rates of progression-free survival (PFS) **(A, B)** and time to next treatment (TTNT) **(C)** in patients with multiple myeloma treated with autoHSCT. Patients are grouped based on the frequency of NKG2A-CD57+ NK cells on day 30 **(A)** and day 100 **(B, C)** after autoHSCT. Patients were grouped based on 66^th^ percentile: high group (>66^th^ percentile) and middle/low group (≤66^th^ percentile).

Then, we carried out survival analysis with the dichotomized phenotypic variables and we observed statistically significant differences in terms of PFS when the frequency of terminally differentiated NKG2A-CD57+ NK cells at S3 and S4 was taken into account. In addition, we also observed differences in terms of TTNT when the frequency of NKG2A-CD57+ NK cells at S4 was analyzed. Specifically, we noticed higher PFS in patients in the middle/low percentile group of NKG2A-CD57+ NK cells at S3 and at S4: median PFS 44.4 *vs* 26.6 months in both S3 (HR=0.45; 95% CI=0.20-0.99; p=0.042), (**Figure 5A**) and S4 (HR=0.36; 95% CI=0.15-0.86; p=0.016) (**Figure 5B**). Furthermore, patients in the middle/low percentile group of NKG2A-CD57+ NK cells also showed a longer TTNT at S4; median TTNT 49.2 *vs* 37.2 months (HR=0.41; 95% CI=0.17-0.98; p=0.039) (**Figure 5C**). Therefore, these data suggest that the differentiation and maturation status of NK cells at +30 and +100 days after autoHSCT affects the clinical outcome of MM patients and that a lower frequency of mature and terminally differentiated NK cells is associated with protection against disease progression after autoHSCT in these patients.

## Discussion

In this study, we have examined the reconstitution of NK cells in patients with MM who had undergone autoHSCT by analyzing different NK cell subsets, their phenotype and functionality, in order to determine an association with the clinical outcome post-autoHSCT. We have demonstrated that there is a redistribution of NK cell subsets and that NK cells express an immature phenotype early after transplantation (S2). Moreover, NK cells seem to be completely functional and present an elevated proliferation capacity. We have documented significantly elevated IL-15 plasma levels when patients reached >1000 leucocytes, that correlate with the number, proliferation capacity and cytotoxic potential of NK cells at this time point. Importantly, to our knowledge, we have revealed a previously undescribed association between the differentiation degree of NK cells at 30 and 100 days after autoHSCT and the clinical outcome of MM patients.

In line with previous studies, both in autoHSCT and alloHSCT settings (17, 24, 25), our data points out to a redistribution of NK cell subsets during their reconstitution after autoHSCT, as observed by a higher frequency of CD56^bright^ NK cells and a lower frequency of CD56^dim^ NK cells, shortly after leukocyte reconstitution. This is also in accordance with the proposed linear model of NK cell development, in which CD56^dim^ are generated from CD56^bright^ NK cells (13). When adaptive NK cells were analyzed, we found that FcϵRγ- NK cells were more abundant than the ones expressing NKG2C and that there were differences between the reconstitution of both cell subsets. The expansion of adaptive NK cells expressing the activating receptor NKG2C has been largely associated with CMV infection or reactivation (7) and with the clinical outcome after autoHSCT and alloHSCT (9, 10). Apart from expressing NKG2C, CMV infection-induced adaptive NK cells have been described to show, among others, high expression of killer-cell immunoglobulin like receptors (KIRs), reduced expression of NKG2A and a lack of expression of PLZF, SYK and EAT2 (26). Moreover, expansion of FcϵRγ- adaptive NK cells related to CMV infection has also been described (27). Nevertheless, although the lack of FcϵRγ is often associated with NKG2C expression, occasionally, these markers may appear disassociated (28, 29) and FcϵRγ- NK cells have been found in CMV seronegative individual as well (27, 30). Thus, the expansion of NKG2C+ NK cells might be more specific to human CMV infection or reactivation than the expansion of FcϵRγ- NK cells (31). The incidence of CMV reactivation after autoHSCT is significantly lower than in alloHSCT (32, 33). Therefore, the notion of adaptive NK cells as a heterogeneous population of cells (26, 29) may explain the dissimilarities that the different subsets showed in terms of their frequency and reconstitution-time needed after autoHSCT.

Consistent with the increment in the percentage of the less differentiated NK cell subset, we also observed an increase in the frequency of NKG2A+ cells and a decrease in the frequency of cells expressing CD57 at leukocyte recovery. This shift in the maturation status of NK cells is maintained until 3 months after autoHSCT, when pre-transplant levels are recuperated. Although our data differ from the increment of the percentage of CD57+ NK cells at leucocyte recovery that Jacobs et al. have previously described (17), they are in accordance with the linear model of NK cell development and how the expression of NKG2A and CD57 is modulated during NK cell maturation (15, 34, 35). In addition, as Jacobs and colleagues observed in their study (17), we also found a surprising expression of CD57 in CD56^bright^ NK cells shortly after autoHSCT that was not observed at any other time point. It is known that CD56^dim^ NK cells are able to upregulate CD56 upon IL-15 stimulation (36) and, as we demonstrated, the IL-15 plasma levels are highly incremented at leucocyte recovery. Thus, it is possible that these cells are CD56^dim^ NK cells that have upregulated CD56. Clearly, more studies are required.

Our data demonstrate an important and previously undescribed link between the NK cell degree of differentiation and the clinical outcome after autoHSCT of MM patients, as observed by the detrimental effect that higher frequency of NKG2A-CD57+ NK cells at +30 days and +100 days post-autoHSCT have in the PFS and TTNT of these patients. Although it is well known that CD57+ NK cells exhibit a more mature phenotype and a higher CD16 induced-cytotoxic capacity, they also display a decreased responsiveness to IL-12 and IL-18 stimulation and a reduced proliferative capacity compared to NK cells lacking CD57 expression (15, 16). High frequencies of tumor infiltrating CD57+ NK cells have been associated with better clinical outcomes in different types of cancer patients (37), probably due to their enhanced cytotoxicity. However, in our study we look at circulating NK cells instead of intratumoral NK cells. And in line with our results, an inverse correlation between circulating CD57+ and tumor-infiltrating NK cells numbers was described in breast cancer patients. These authors also found that patients with higher numbers of circulating CD57+ NK cells have lower pathological complete response rates to early treatment with anti-HER2 therapeutic antibodies (38). In a similar manner, other studies have described an accumulation of CD57+CD8+ T cells in patients with different types of cancers and also an association between high frequencies of circulating CD57+CD8+ terminally differentiated T cells and poor prognosis (37, 39, 40). On the other hand, it is known that CD57+ NK cells have a lower expression of the CXCR4 chemokine receptor, which is involved in the homing to the bone marrow, than CD57- NK cells (15). Therefore, this could also be another mechanism contributing to the poor prognosis of patients with a higher frequency of circulating terminally differentiated NK cells, where less NK cells home to the bone marrow. Nevertheless, the above mentioned studies did not analyzed the co-expression of NKG2A and CD57, and thus within those CD57+ NK cells there would be NKG2A+ and NKG2A- NK cells that might have different functional and proliferative capacities. Therefore, our results, in which we identified an association between the NKG2A-CD57+ NK cell subset and disease progression, adds new information about a specific NK cell subset with an important role in MM patients undergoing autoHSCT.

IL-15 is a critical cytokine for NK cell differentiation, survival and proliferation (22, 41). Monocytes and dendritic cells have been described as a source of this cytokine (42). In NHL patients, IL-15 has been proposed to influence clinical outcome through NK cell recovery after autoHSCT (23). In contrast to Porrata et al., not only we did not observed any association between IL-15 plasma levels and clinical outcome, but we also found a statistically significant negative correlation between IL-15 plasma levels at S2 and absolute NK cells numbers. However, our cohort of patients is composed by MM patients whereas Porrata et al. described those results in NHL patients. On the other hand, and similar to our results, a strong inverse correlation between NK cell numbers and plasma IL-15 levels at 2 weeks post-transplantation was observed in allo-HSCT (36). The elevated plasma levels found early after autoHSCT might be related to transplant-regimen induced depletion of lymphoid cells that consume circulating IL-15. Considering that IL-15 production is tightly regulated (42), it is tempting to speculate that in those situations with high plasma IL-15 levels, this is due to the existence of a low number of cytokine-consuming cells, among which are NK cells. When the number of these cells increase, the plasma levels of IL-15 decrease to almost undetectable levels.

Our data showed that NK cells experience a shift in terms of, among others, cytotoxic potential and proliferation capacity at leucocyte recovery. The percentage of Ki67+ cells is dramatically increased in the three NK cell subsets and the expression of granzyme B is incremented in CD56^bright^ NK cells. It is very likely that these changes are due to the particular cytokine milieu that is present at that specific time. The role of IL-15 in upregulating Ki67 expression on NK cells has been previously described (43, 44). On the other hand, while at rest CD56^bright^ NK cells display a low expression of granzyme B, after an IL-15 priming granzyme B protein levels significantly increase in this subset (45).

To our knowledge, this is the first study that performed such extended analysis of NK cell phenotype in MM patients after autoHSCT and that includes other unconventional NK cell subsets, such as CD56^neg^ and several adaptive NK cell subsets. Furthermore, unlike previous studies in autoHSCT in which NK cells were only examined up to 1 month after autoHSCT (17), our analysis goes further by analyzing samples up to 1 year after transplantation. In conclusion, we were able to demonstrate that there is a redistribution of NK cells subsets and a shift in NK cells phenotype shortly after autoHSCT. Importantly, we observed that MM patients with lower frequency of mature NK cells, identified by the NKG2A-CD57+ phenotype, showed better clinical outcomes after autoHSCT. Although more studies in a larger cohort of patients are needed to validate these results, our data provide new insight into the importance of NK cell reconstitution and their degree of differentiation that could help to improve outcomes after autoHSCT in MM patients.

## Data Availability Statement

The raw data supporting the conclusions of this article will be made available by the authors, without undue reservation.

## Ethics Statement

The studies involving human participants were reviewed and approved by Basque Ethics Committee for Clinical Research. The patients/participants provided their written informed consent to participate in this study.

## Author Contributions

FB and OZ conceived the project. FB and AO designed experiments. CG, AU, JM-M, and JG-R obtained the clinical samples and clinical data from patients. AO performed experiments. MRi and MRe performed NK cell number determination. AO and SP-F performed data analysis. AO designed figures. FB, IT, GA-P, and OZ provided intellectual input. FB and AO wrote the manuscript. All authors critically reviewed the manuscript. All authors contributed to the article and approved the submitted version.

## Funding

Supported by the following grants: AECC-Spanish Association Against Cancer (PROYE16074BORR) and Health Department, Basque Government (2020333024). AO and GA-P are recipient of a fellowship from the Jesús de Gangoiti Barrera Foundation (FJGB20/007 and FJGB20/002). IT is recipient of a predoctoral contract funded by the Department of Education, Basque Government (PRE_2020_2_0007). GA-P is recipient of a predoctoral contract funded by AECC-Spanish Association Against Cancer (PRDVZ21440ASTA). OZ is recipient of a postdoctoral contract funded by “Instituto de Salud Carlos III-Contratos Sara Borrell 2017 (CD17/00128)” and the European Social Fund (ESF)-The ESF invests in your future. FB is an Ikerbasque Research Professor, Ikerbasque, Basque Foundation for Science.

## Conflict of Interest

The authors declare that the research was conducted in the absence of any commercial or financial relationships that could be construed as a potential conflict of interest.

## Publisher’s Note

All claims expressed in this article are solely those of the authors and do not necessarily represent those of their affiliated organizations, or those of the publisher, the editors and the reviewers. Any product that may be evaluated in this article, or claim that may be made by its manufacturer, is not guaranteed or endorsed by the publisher.
