# Supplementary material for: NK Cell Reconstitution After Autologous Hematopoietic Stem Cell Transplantation: Association Between NK Cell Maturation Stage and Outcome in Multiple Myeloma
Source: Front Immunol. 2021 Oct 5;12:748207. doi: 10.3389/fimmu.2021.748207 (PMC8524090; doi:10.3389/fimmu.2021.748207)
Supplement: Supplementary file 1 [file DataSheet_1.pdf]

Supplementary Material

This file includes:

1. Supplementary Figure 1: Gating strategy for the identification of NK cells.
2. Supplementary Figure 2. NK cell subset distribution is not affected by the time to leukocyte recovery.
3. Supplementary Figure 3. NK cells are functional shortly after autoHSCT.
4. Supplementary Figure 4. Correlation of IL-15 plasma levels and flow cytometry data in sample S2.
5. Supplementary Table 1. Flow cytometry panels.

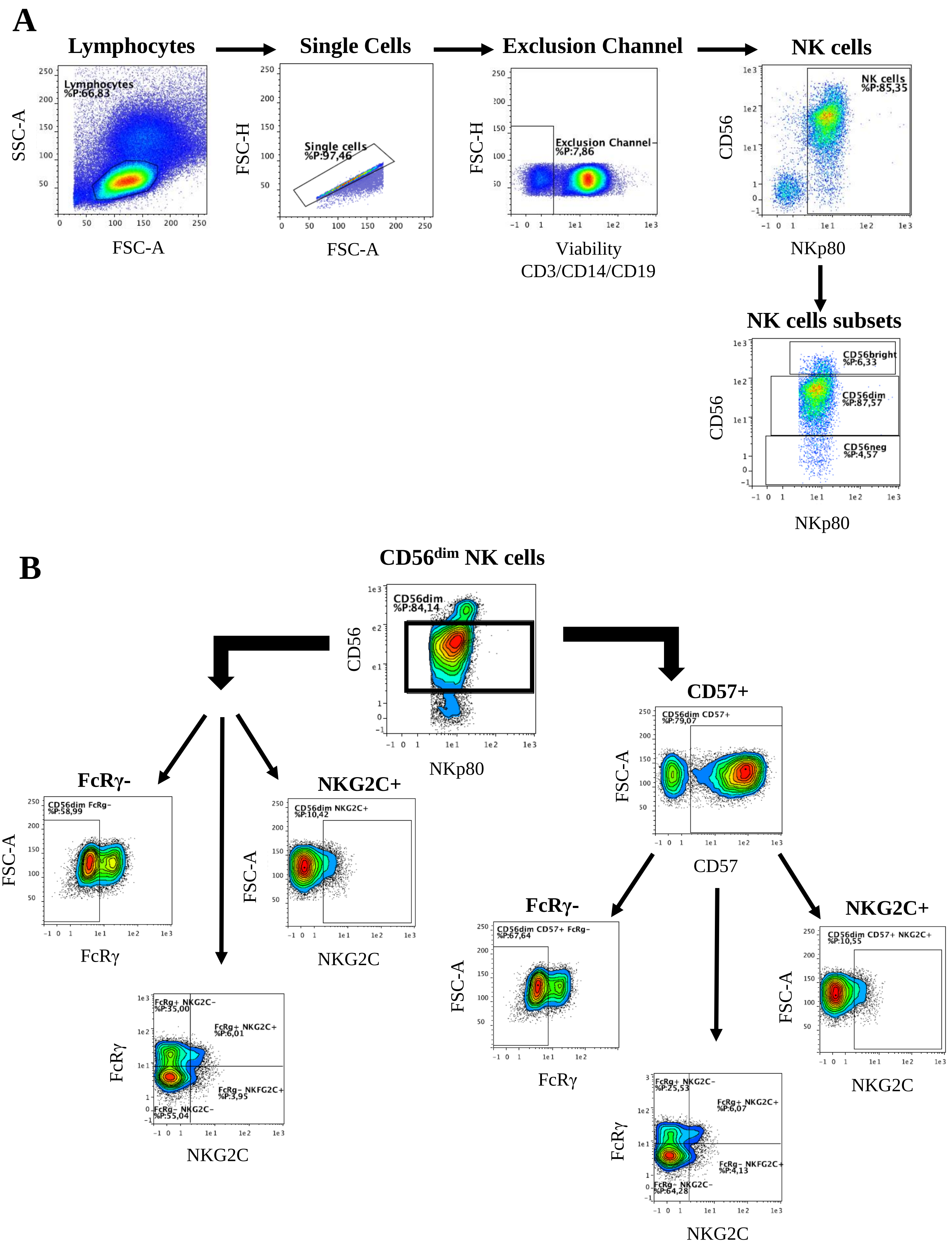

A
Lymphocytes
Single Cells
Exclusion Channel
NK cells
Lymphocytes %P:66,83
Single cells %P:97,46
Exclusion Channel- %P:7,86
NK cells %P:85,35
SSC-A
FSC-A
FSC-H
Viability CD3/CD14/CD19
CD56
NKp80
NK cells subsets
CD56bright %P:6,33
CD56dim %P:87,57
CD56neg %P:4,57
B
CD56dim NK cells
CD56dim %P:84,14
CD57+
CD56dim CD57+ %P:79,07
CD57
FcRγ-
CD56dim FcRg- %P:58,99
FcRγ
NKG2C+
CD56dim NKG2C+ %P:10,42
NKG2C
FcRg+ NKG2C- %P:35,00
FcRg+ NKG2C+ %P:6,01
FcRg- NKFG2C+ %P:3,95
FcRg- NKG2C- %P:55,04
CD56dim CD57+ FcRg- %P:67,64
CD56dim CD57+ NKG2C+ %P:10,55
FcRg+ NKG2C- %P:25,53
FcRg+ NKG2C+ %P:6,07
FcRg- NKFG2C+ %P:4,13
FcRg- NKG2C- %P:64,28
FSC-A

Supplementary Figure 1. **Gating strategy for the identification of NK cells**. (A) Pseudocolor plot graphs representing the gating strategy utilized for the identification of NK cells and NK cell subsets. Data from a representative patient is shown. Lymphocytes were electronically gated based on their forward and side scatter parameters and then single cells were selected. To identify NK cells, the population negative for the exclusion channel (viability, CD3, CD14 and CD19) was selected. After that $CD56^{bright}$, $CD56^{dim}$ and $CD56^{neg}$ NK cell subsets were identified based on the CD56 and NKp80 markers. Finally, the expression of different markers were analyzed within total NK cells or NK cell subsets. (B) Pseudocolor and contour plot graphs representing the gating strategy utilized for the identification of adaptive NK cells. Data from a representative patient is shown. The frequency of the different adaptive NK cells was analyzed within the $CD56^{dim}$ NK cell subset. The individual expression of FcRγ and NKG2C in addition to the co-expression of both markers was analyzed within $CD56^{dim}$ NK cells (left) and within $CD56^{dim}$CD57+ NK cells (right).

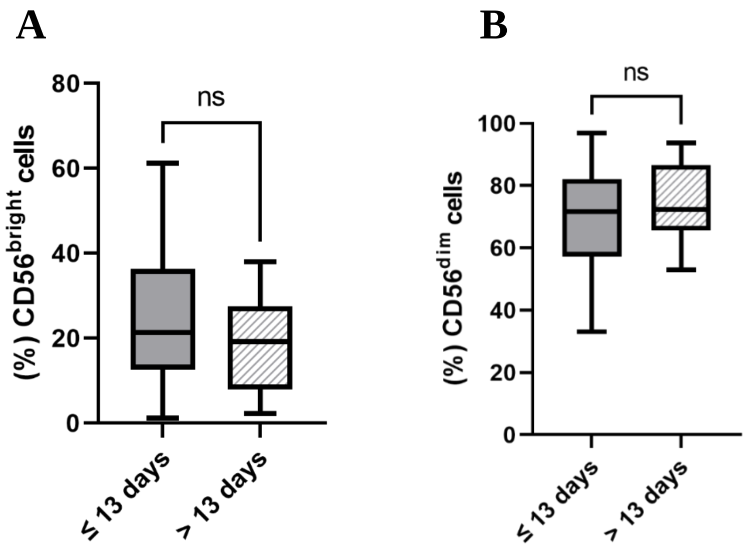

Supplementary Figure 2. **NK cell subset distribution is not affected by the time to leukocyte recovery**. Boxplot graphs showing the percentage of CD56^{bright} (A) and CD56^{dim} (B) in sample S2 from patients with a time period of ≤13 days or >13 days between autoHSCT and S2. Boxplots show the median and 25–75th percentiles, and the whiskers denote lowest and highest values. ns: no significant..

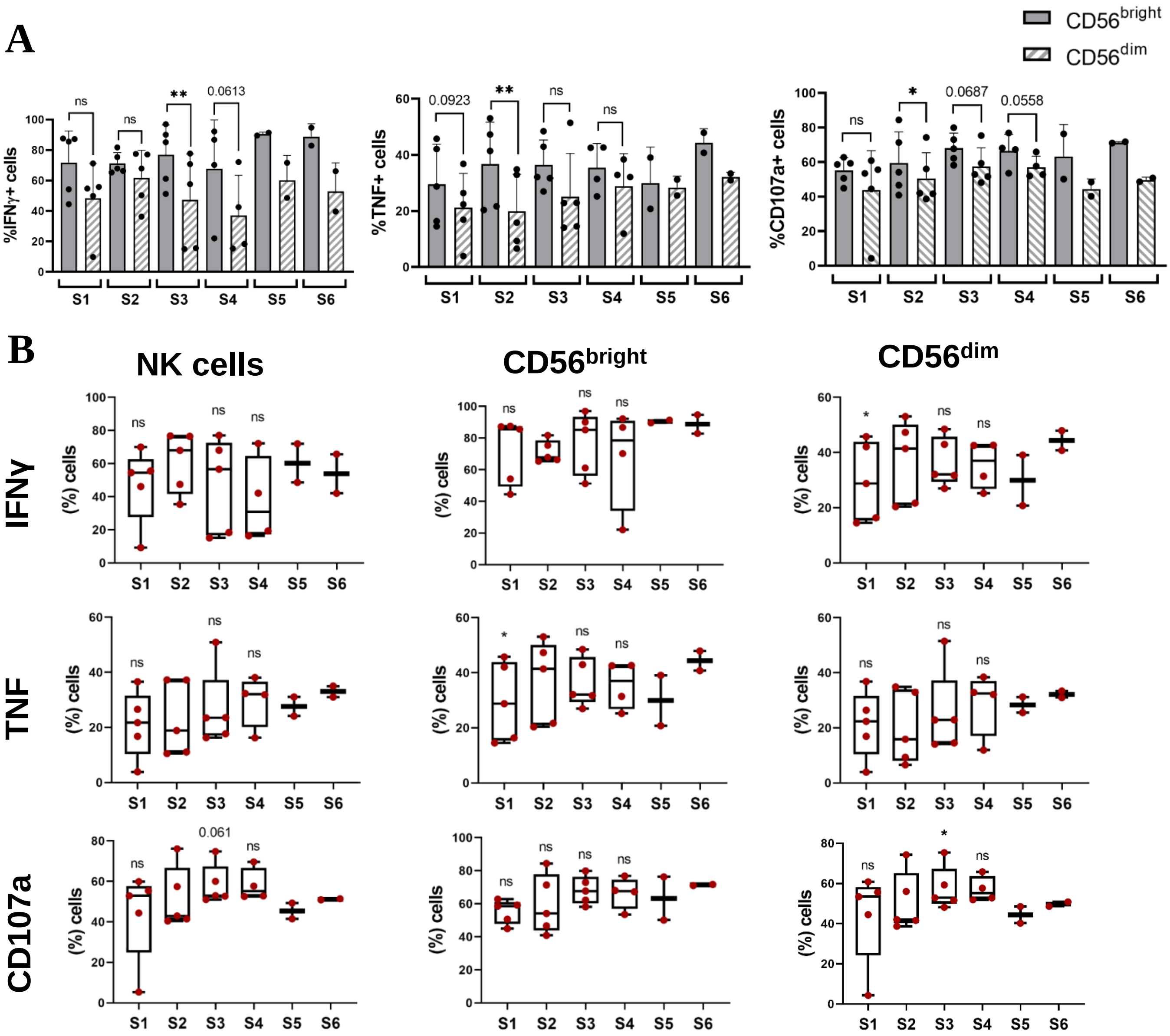

Supplementary Figure 3. NK cells are functional shortly after autoHSCT. (A) Bar graphs showing the percentages of IFNγ+, TNF+ and CD107a+ cells within $CD56^{bright}$ and $CD56^{dim}$ NK cell subsets at the six studied time points (S1-S6). (B) Boxplot graphs showing the percentage of IFNγ+, TNF+ and CD107a+ cells within total NK cells and $CD56^{bright}$ and $CD56^{dim}$ NK cell subsets at the six studied time points (S1-S6). The production of IFNγ by NK cells was measured after stimulation with IL-12+IL-15+IL-18 for 25-26 hours. The production of TNF and degranulation (CD107a) of NK cells were measured after stimulation with 721.221 cell line for 5 hours. The percentage of NK cells positive for IFNγ, TNF and CD107a was calculated after subtracting the non-stimulus condition. Bar graphs show the mean with standard deviation (SD). Boxplots show the median and 25–75th percentiles, and the whiskers denote lowest and highest values. Each dot represent a donor. Due to the low number of cells we had from each patient, especially at S2, complete functional analysis of all the samples was only possible in one patient. However, for our analysis, we also included patients in which functional analysis was carried out at least in the first three samples (S1, S2, S3) (n=5) *$p<0.05$, **$p<0.01$ and ns: no significant.

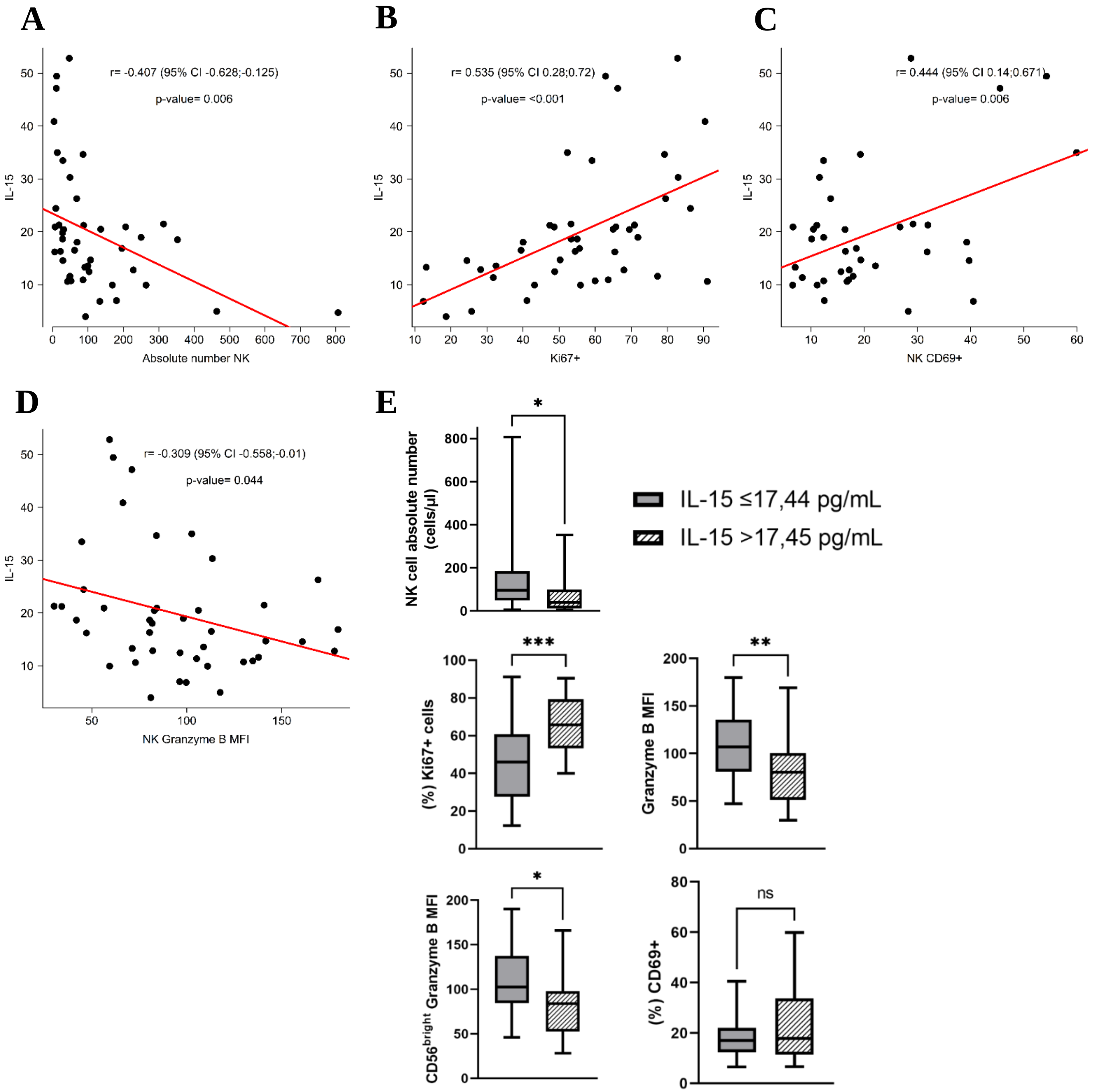

Supplementary Figure 4. Correlation of IL-15 plasma levels and flow cytometry data in sample S2. Correlation plots showing association between IL-15 plasma levels at S2 and the absolute number of NK cells (A), the percentage of Ki67+ (B) and CD69+ (C) NK cells and the media fluorescence intensity (MFI) of granzyme B within NK cells at S2. (E) Boxplot graphs showing the NK cell absolute number (cells/µl), percentage of Ki67+ within total NK cells, MFI of granzyme B within total NK cells and within CD56^{bright} NK cells and the percentage of CD69+ cells within total NK cells of the sample S2 in patients with IL-15 plasma levels of ≤17.44 pg/mL or >17,45 pg/mL (median IL-15 levels at S2: 17.45 pg/mL). Boxplots show the median and 25–75th percentiles, and the whiskers denote lowest and highest values. *p<0.05, **p<0.01, ***p<0.001, and ns: no significant.

Supplementary Table 1. Flow cytometry panels

Phenotype panel 1

Laser	Filter	Fluorochrome	Marker	Manufacturer	Clone
405	450/50	BV421	CD56	BD Bioscience	NCAM 16.2
	525/50	BV510	CD3/CD14/CD19	BD Bioscience	UCHT1/ MφP9/ SJ25C1
	525/50	Aqua Dead	Viability	Invitrogen	
488	525/50	FITC	FcεRγ	Merck	
	585/40	PE	NKG2C	R&D systems	134591
	655-730				
	750LP	PEVio770	NKp80	Miltenyi Biotec	4A4.D10
635	655-730	APC	NKG2A	Beckman Coulter	Z199
	750LP	APCVio770	CD57	Miltenyi Biotec	REA769

Phenotype panel 2

Laser	Filter	Fluorochrome	Marker	Manufacturer	Clone
**405**	450/50	BV421	CD56	BD Bioscience	NCAM 16.2
	525/50	BV510	CD3/CD14/CD19	BD Bioscience	UCHT1/ MφP9/ SJ25C1
	525/50	Aqua Dead	Viability	Invitrogen	
**488**	525/50	FITC	Ki67	Invitrogen	20Raj1
	585/40	PE	Granzyme B	BD Bioscience	GB11
	655-730				
	750LP	PEVio770	NKp80	BD Bioscience	4A4.D10
**635**	655-730				
	750LP	APCVio770	CD69	Miltenyi Biotec	REA824

Functional panel

Laser	Filter	Fluorochrome	Marker	Manufacturer	Clone
**405**	450/50	BV421	CD56	BD Bioscience	NCAM 16.2
	525/50	BV510	CD3/CD14/CD19	BD Bioscience	UCHT1/ MφP9/ SJ25C1
	525/50	Aqua Dead	Viability	Invitrogen	
**488**	525/50				
	585/40	PE	CD107a	Miltenyi Biotec	REA792
	655-730	PerCP-Cy5.5	IFNγ	BD Biosciences	B27
	750LP	PEVio770	NKp80	BD Bioscience	4A4.D10
**635**	655-730	APC	TNF	BioLegend	MAb11
	750LP				
